# Severe Human Granulocytic Anaplasmosis With Significantly Elevated Ferritin Levels in an Immunocompetent Host in Pennsylvania: A Case Report

**DOI:** 10.1177/2324709618758350

**Published:** 2018-02-13

**Authors:** Mona L. Camacci, Ronaldo Paolo Panganiban, Zachary Pattison, Kamyar Haghayeghi, Alexander Daly, Cindy Ojevwe, Ryan J. Munyon

**Affiliations:** 1 Penn State Milton S. Hershey Medical Center, Hershey, PA, USA

**Keywords:** human granulocytic anaplasmosis, hyperferritinemia, hemophagocytic lymphohistiocytosis

## Abstract

Human granulocytic anaplasmosis (HGA) is a tick-borne, infectious disease caused by *Anaplasma phagocytophilum* that generally presents with nonspecific symptoms such as fever, chills, headache, malaise, and myalgia. If not treated immediately, HGA can cause hemophagocytic lymphohistiocytosis (HLH), a well-documented but underrecognized sequela of severe HGA. In this article, we report a case of severe HGA with hyperferritinemia in a 74-year-old male from Central Pennsylvania who initially presented with recurrent fevers, nausea, and malaise to our emergency department and was subsequently discharged home that same day. Ten days later, the patient returned with acute kidney injury, elevated liver transaminases, and profound hyperferritinemia to 5130 ng/mL. Empiric doxycycline was administered for suspected tick-borne disease and serologies eventually came back positive for anti–*Anaplasma phagocytophilum* antibodies. The patient returned to baseline status 15 days after discharge. Our case shows the challenges in the timely diagnosis of HGA and highlights the role of serum ferritin in aiding this diagnosis. Although our patient did not fulfill the HLH diagnostic criteria, our report demonstrates the importance of recognizing HGA as a reversible cause of HLH.

## Introduction

Human granulocytic anaplasmosis (HGA) is a tick-borne disease caused by *Anaplasma phagocytophilum.* Transmission occurs via ticks of the *Ixodes* genus.^1,2^ These ticks act as a vector for several other disease-causing viruses, bacteria, and parasites, including; Ehrlichia,^3^ *Rickettsia*,^4^ *Borrelia*,^5^ *Babesia*,^6,7^ and Powassan virus.^8^ HGA generally presents with nonspecific symptoms such as fever, chills, headache, malaise, and myalgia.^9^ Typical laboratory findings include leukopenia, thrombocytopenia, and elevated transaminases.^10^ Anaplasmosis is a potentially fatal disease for immunocompromised patients who remain untreated. Most patients will experience resolution of symptoms within 48 hours of treatment with doxycycline. Majority of patients with anaplasmosis do not require hospitalization, and the overall case fatality rate for this disease is 0.3%.^11^ The highest incidence of infection occurs in June and July, most often affecting patients aged 60 years or older.^11^

Ferritin is an important iron storage protein that has been implicated in several important biological process including iron homeostasis, cell-mediated immunity, and inflammation.^12^ Ferritin is an acute phase reactant and marker of inflammation.^13^ Significant serum ferritin elevation (>5000 ng/mL) is associated with a narrow differential diagnosis, which includes sepsis, primary and secondary hemophagocytic lymphohistiocytosis (HLH), liver disease, solid or hematologic malignancy, iron overload secondary to chronic transfusions, adult-onset Still’s disease, and hemophagocytic syndrome.^14-17^ Accurate diagnosis of illnesses associated with hyperferritinemia is essential as many of these diseases may prove fatal if left untreated.

In this report, an otherwise healthy individual not residing in an area historically considered endemic for HGA presented with profound hyperferritinemia, acute kidney injury (AKI), transaminitis, hyperbilirubinemia, normocytic anemia, and hyponatremia. This individual was diagnosed with anaplasmosis confirmed by laboratory testing with possible early *Borrelia burgdorferi* and Epstein-Barr virus (EBV) infection. The patient rapidly improved with antibiotic therapy, thereby obviating the need for further testing for HLH resulting in patient not fulfilling the HLH diagnostic criteria.^18^

## Case Description

A 74-year-old Caucasian male presented to the hospital in late June after evaluation by his primary care physician (PCP) for hematuria and nausea. His medical history included hypertension, hyperlipidemia, gout, and hypothyroidism. On presentation to the emergency department (ED), the patient was hemodynamically stable and afebrile, with an elevated creatinine of 1.4 mg/dL. Although the patient’s baseline creatinine was unknown, review of the electronic medical record revealed a creatinine of 1.09 mg/dL several years prior (normal = 0.70-1.30 mg/dL). Urinalysis revealed moderate hemoglobinuria.

Initial laboratory evaluation showed a normal leukocyte count of 6.9 × 10^9^ cells/L (normal = 4.0-10.4 × 10^9^ cells/L) and normal liver function with a bilirubin of 0.7 mg/dL (normal = 0.2-1.3 mg/dL), alanine transaminase (ALT) 42 U/L (normal = 13-69 U/L), aspartate aminotransferase (AST) 38 U/L (normal = 15-46 U/L), and alkaline phosphatase 70 U/L (normal = 38-126 U/L). He was discharged home with outpatient follow-up scheduled. He returned to the ED 5 days later with nightly fevers. A computed tomography of the abdomen and pelvis showed diverticulosis, a 5-mm nodule in the right lower lobe of the lung, hiatal hernia, small fat-containing umbilical hernia, and enlarged prostate. On repeat laboratory evaluation, he was found to have a creatinine of 1.6 mg/dL, an ALT of 84 U/L (normal 13-69 U/L), and a sodium of 132 mmol/L (normal = 137-145 mmol/L). He was again discharged home to follow-up with his PCP. Outpatient laboratory values obtained days later demonstrated worsening AKI with creatinine 1.9 mg/dL. He also had worsening liver function AST 128 U/L, ALT 166 U/L, and total bilirubin of 3.9 mg/dL. At that time, his erythrocyte sedimentation rate was 58 mm/h (normal = 0-40 mm/h). The patient was referred back to the ED by his PCP for evaluation of recurrent fevers and worsening AKI. Because of his worsening clinical status, the patient was admitted to our Internal Medicine service.

On admission, the patient was febrile to 38.8°C, but otherwise hemodynamically stable. Laboratory values demonstrated serum creatinine 2.2 mg/dL, white blood cells 11 × 10^9^ cells/L, hemoglobin 10.8 g/dL (normal = 13.0-17.0), platelets 96 × 10^9^ cells/L (normal = 150-350 × 10^9^ cells/L), ALT 186 U/L, AST 148 U/L, total bilirubin 6.6 mg/dL, γ-glutamyl transferase 310 U/L (normal = 12-58 U/L), and alkaline phosphatase 298 U/L. Ferritin was 5130 ng/mL (normal = 17.9-464.0 ng/dL). The patient initially received broad spectrum antibiotic therapy with piperacillin/tazobactam and vancomycin on admission. Doxycycline was added the morning following admission for empiric coverage of tick-borne diseases. Ferritin levels decreased to 4450 ng/dL the day after doxycycline and to 1750 ng/dL 5 days after initiation of doxycycline therapy. The patient reported feeling better with mild nausea and improved appetite. Antibiotics were deescalated to doxycycline monotherapy. The patient remained afebrile. Five days following admission, he was discharged home to complete a 10-day course of doxycycline. Fifteen days after initial presentation, ferritin levels were trending downwards at 867 ng/dL.

## Microbiological Testing

**Figure 1.**
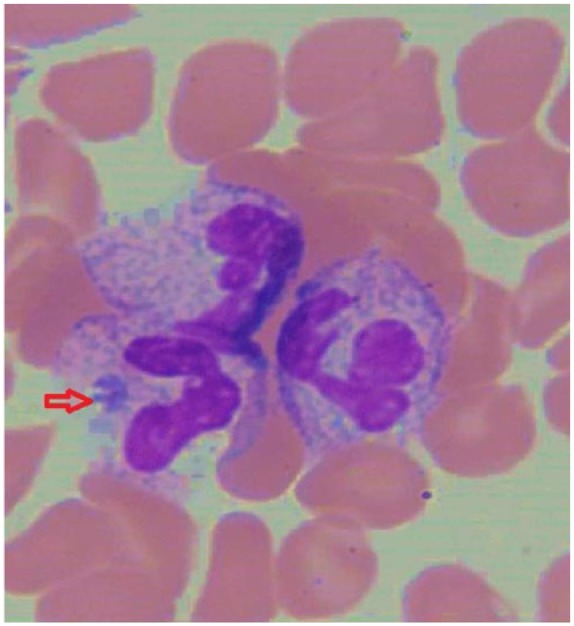
Peripheral smear from hospital day 2 showing rare morula inclusion in a neutrophil consistent with *Anaplasma phagocytophilum* in a patient with positive antibody titers. Patient received doxycycline treatment for 1 day when this smear was obtained.

Peripheral smear showed morula inclusions in neutrophils (Figure 1). Laboratory results confirmed acute anaplasmosis infection by serology with positive titers for IgM antibodies of 1:256 (normal = <1:16) and IgG antibodies of 1:256 (normal = <1:80) by immunofluorescence (Associated Regional and University Pathologists, Salt Lake City, UT). Lyme IgM antibodies were positive but Lyme IgG antibodies were negative indicating potential early *Borrelia* infection. There was no evidence of acute coinfection by either *Babesia* or *Ehrlichia chaffeensis.* Quantitative serum EBV PCR (polymerase chain reaction) demonstrated co-infection with EBV at 851 copies/mL (normal <500 copies/mL).

## Discussion

Incidence of HGA has increased from 348 reported cases in 2000 to approximately 1800 in 2010.^19^ In the United States of America, HGA mostly occurs in Minnesota, Wisconsin, and New York.^11^ Our patient is from Central Pennsylvania with no known travel to endemic areas of HGA. He denied any recent tick bites. The lack of clear exposure history likely contributed to the delay in the patient’s diagnosis. Nonetheless, from 2008 to 2012, there were 19 reported cases of HGA in Pennsylvania.^11^ The deer tick, *Ixodes scapularis*, is found in all 67 counties of Pennsylvania and is the primary vector for *A phagocytophilum* in the American Northeast. Indeed, approximately 3.3% of *I scapularis* in Pennsylvania carry *A phagocytophilum*.^20^ Given the increasing HGA incidence and the wide availability of its vector, familiarity with the disease and its clinical presentation would allow for timely management with minimal complications.

Most patients with HGA experience a subclinical illness; however, a variety of clinical presentations are possible.^21^ However, symptomatic individuals typically present with fever, myalgia, headaches, and nonspecific gastrointestinal or respiratory symptoms.^22^ Our patient initially complained of nausea and fever. His nausea improved with ondansetron on initial presentation to the ED. He was clinically stable and was discharged home. Five days later, our patient returned to the ED with persistent fever and nausea. This time, his liver function tests showed mild ALT and AST elevations. He also exhibited mild anemia and hyponatremia. Because our patient remained stable and his laboratory values presented nonspecific findings, he was again discharged home. The patient’s nonspecific symptoms and laboratory values clearly presented a challenge in making a diagnosis. This, combined with a low clinical suspicion for HGA, contributed to the delay in our patient’s treatment.

Typically, laboratory values from patients with HGA reveal leukopenia, thrombocytopenia, and transaminitis.^22^ Diagnosis depends on a high index of suspicion. Serum antibodies are usually not detectable until 4 days after onset.^23^ In symptomatic patients, lack of early treatment can result in severe disease and hospitalization, especially among immunocompromised and elderly individuals. Patients with severe disease can experience AKI, acute respiratory distress syndrome, meningitis, encephalitis, pneumonia, disseminated intravascular coagulation, and sepsis.^11^

Detection of *A phagocytophilum* can occur in many ways. These include PCR, peripheral smear, and immunohistochemistry. Culturing is also possible but its sensitivity has been shown to be similar to that of PCR and peripheral smear. PCR has the highest sensitivity and specificity with a rapid turnaround time, which makes it the most frequently used method of confirming anaplasmosis. Blood must be obtained before treatment or within 48 hours of antibiotic therapy. Detection of morulae by peripheral smear is seen in 25% to 75% of infected patients who have not begun treatment (Figure 1). Peripheral smear sensitivity is highest during the first week of infection.^24^ In the unfortunate event that a patient fatally succumbs to anaplasmosis, immunohistochemistry can be helpful with tissue obtained during autopsy.

Another well-established but underrecognized complication of HGA is HLH.^25,26^ Characterized by macrophage and T-cell overactivation, HLH results in excessive cytokine production that produces a hyperinflammatory milieu, which can lead to tissue destruction.^18^ Diagnosis of HLH is based on fulfilling 5 of the 8 findings: fever; splenomegaly; peripheral blood cytopenia; hypertriglyceridemia; hemophagocytosis in the bone marrow, spleen, or lymph node; low or absent natural killer (NK) cell activity; hyperferritinemia; and elevated soluble IL-2 receptor.^18^

The molecular mechanisms by which *A phagocytophilum* infection results in HLH remain incompletely understood. Studies have shown that *A phagocytophilum* employs several immune evasion strategies including avoidance of granulocyte phagocytosis and respiratory burst.^27,28^ However, it is unclear how these immune evasion mechanisms result in severe disease. NK cells, NK T-cells, and cytotoxic T-cells have also been implicated in the development of severe inflammatory phenotypes associated with anaplasmosis.^29^

Hyperferritinemia, a key characteristic of HLH, may be derived from *A phagoctyphilum*–induced upregulation of ferritin expression in neutrophils.^30^ Furthermore, Carlyon et al showed that increased ferritin expression during the initial hours of *A phagocytophilum* infection of human cells in vitro occurs as a response to reactive oxygen species production during bacterial binding and invasion.^30^ These mechanisms may account for the hyperferritinemia observed in HGA-associated cases of HLH. Indeed, levels of serum ferritin has been shown to correlate with severity of HGA.^25^ Looking ahead, we recommend obtaining ferritin levels in patients thought to have a possible anaplasmosis. Although ferritin is not specific for anaplasmosis, its elevation in the appropriate clinical context could aid in the diagnosis of anaplasmosis.

Other potential causes of hyperferritinemia in our patient are EBV infection and Lyme disease. Several clinical reports have consistently shown EBV as a virus associated with HLH-induced hyperferritinemia.^31^ Majority of these cases however were from Asia. EBV-associated HLH also typically affect younger individuals and usually have much higher EBV viral load.^32,33^ By contrast, our patient is an older individual of Caucasian descent with no recent travel to Asia and with only a mildly elevated EBV viral load. Most notably, no specific anti-EBV treatment was administered to our patient. As such, it is unlikely that the patient’s hyperferritinemia was because of his EBV infection. Additionally, Lyme disease has been reported as very rare cause of HLH. To our knowledge, only a single report of this phenomenon exists to date; the patient had disseminated Lyme disease with central nervous system involvement and no evidence of other infections.^34^ In comparison, our patient had a positive Lyme IgM but negative IgG, indicating early infection or false-positive test result. Our patient also did not have any evidence of central nervous system involvement. Nonetheless, the biomolecular effects of coinfection with *Borrelia* and *Anaplasma* and the resulting clinical morbidity and mortality are active areas of investigation.^35-37^ Thus far, it remains unclear whether coinfection with these agents results in additive or synergistic disease morbidity.^38-41^

On admission, our patient presented with fever, anemia, thrombocytopenia, and marked hyperferritinemia with no splenomegaly, thereby fulfilling only 3 out 8 of the HLH-2004 criteria. We did not evaluate for presence of hypertriglyceridemia or elevated soluble IL-2 receptor. Hemophagocytosis was not seen on peripheral smear. No tissue or bone marrow samples were obtained to further investigate hemophagocytosis. As such, it is unclear whether or not our patient had HLH. Nonetheless, since being first described in a case series of 29 patients in 2007 by Dumler et al,^25^ HGA-induced HLH has been reported in New York,^42^ South Korea,^43^ and Greece.^44^ It is essential to identify HGA and other tick-borne diseases as an inciting cause of HLH. Absent a triggering etiology, initial treatment for HLH involves 8 weeks of etoposide and dexamethasone.^18^ Other options include cyclophosphamide, vincristine, and doxorubicin.^45,46^ However, patients with bacterial-induced HLH often rapidly respond to antibiotic therapy, thereby obviating the need to treat with chemotherapeutic and immunosuppressive drugs.^42,47^ Although our patient did not fulfill the HLH-2004 criteria, his symptomatology rapidly improved with empiric antibiotics and he was subsequently deescalated to doxycycline monotherapy.
